# Supplementary material for: Exploring Cryptococcus neoformans CYP51 and Its Cognate Reductase as a Drug Target
Source: J Fungi (Basel). 2022 Nov 28;8(12):1256. doi: 10.3390/jof8121256 (PMC9785471; doi:10.3390/jof8121256)
Supplement: Supplementary file 1 [file jof-08-01256-s001.zip › jof-2041593-supplementary.pdf]

Ruma et al Supplemental Materials

Table S1. *Saccharomyces cerevisiae* parental strains used in the study.

Strain	Genotype	Source	Reference
**ADΔ**	MATα *PDR1-3 ΔYOR1::hisG ΔSNQ2::hisG ΔPDR3::hisG ΔPDR10::hisG ΔPDR11::hisG ΔYCF1::hisG ΔPDR55::hisG ΔPDR15::hisG Δura3 Δhis*AD124567 *ΔPDR5::hisG ΔPDR15::hisG, Δura3*	Lamping et al, 2007	[1]
**Y941**	ADΔ, Δpdr5::ScCYP51-6xHIS-URA3	Monk et al, 2014	[2]
**Y1857**	AD2Δ (ADΔ, ΔHIS1::dpl200)	Sagatova et al, 2016	[3]
**Y2411**	AD2Δ, ΔPDR5::URA3	E. Lamping	
**Y2494**	AD2Δ, ΔCYP51pro::GAL1pro	Monk et al, 2019	[4]

Table S2. Oligonucleotides used in the study.

**A. Construction of CpCYP51-6×His Y132F cassette by 3-fragment fusion for expression from the *PDR5* locus**		
1. Amplification of CnCYP51-6×His ORF from plasmids supplied by ATUM		
Forward primer	PDR5us-CnCYP51_f (52)	CCGCTCGTTCGAAAGACTTAATTAAAAAATGT CAGCCATCATCCCTCAAGTG
Reverse primer	6 HisStop_r (26)	CGAATTTAATGATGATGGTGATGATG
2. Amplification of upstream fragments from gDNA of selected strain		
Forward primer	PDR5Fv3_f (23)	TCGCATTCTGCGCCTTCGAGCAC
Reverse primer	pABC3-PacI_r (31)	CATTTTTTAATTAAGTCTTTCGAACGAGCGG
3. Amplification of downstream fragment from gDNA of selected strain		
Forward primer	6HisStop-f (26)	CATCATCACCATCATCATTAAATTCG
Reverse primer	PDR5_186DS_r (25)	TTCGGACATTGAACTTTGATTTATC
**B. Construction of CnCPR-6×His cassette by 3-fragment fusion for expression from the *PDR15* locus**		
1. Amplification of CnCPR-6×His ORF from plasmids received from ATUM		
Forward primer	PDR5us-CnCPR (55)	CCGCTCGTTCGAAAGACTTAATTAAAAAATGC TATCTGCAGTGGACATTGTTATC
Reverse primer	6 HisStop -r (26)	CGAATTTAATGATGATGGTGATGATG
2. Amplification of upstream fragments from gDNA of selected strain		
Forward primer	PDR15us_f (25)	GTCACGCCGCCGAACTGCAGCGCGC
Reverse primer	pABC3-PacI_r (31)	CATTTTTTAATTAAGTCTTTCGAACGAGCGG
3. Amplification of downstream fragment from gDNA of selected strain		
Forward primer	Not1-6xHis (34)	GGCGGCCGCCATCATCACCATCATCATTAAATTC
Reverse primer	PDR15DS_r (25)	GATGGAATAATCCAGTTCGACTCTG
**C. Amplification of ScHIS1 disruption cassette from gDNA of selected strains**		
Forward primer	ScErg11_US-773_f (24)	GCAACAATGGGCGGTTGTTTAGAG
Reverse primer	ScErg11DS346_r (25)	GACTGCTTTATTTCtGCTTGGCCTG

f and r denotes forward and reverse primer respectively. The number of nucleotides in each primer is shown in brackets.

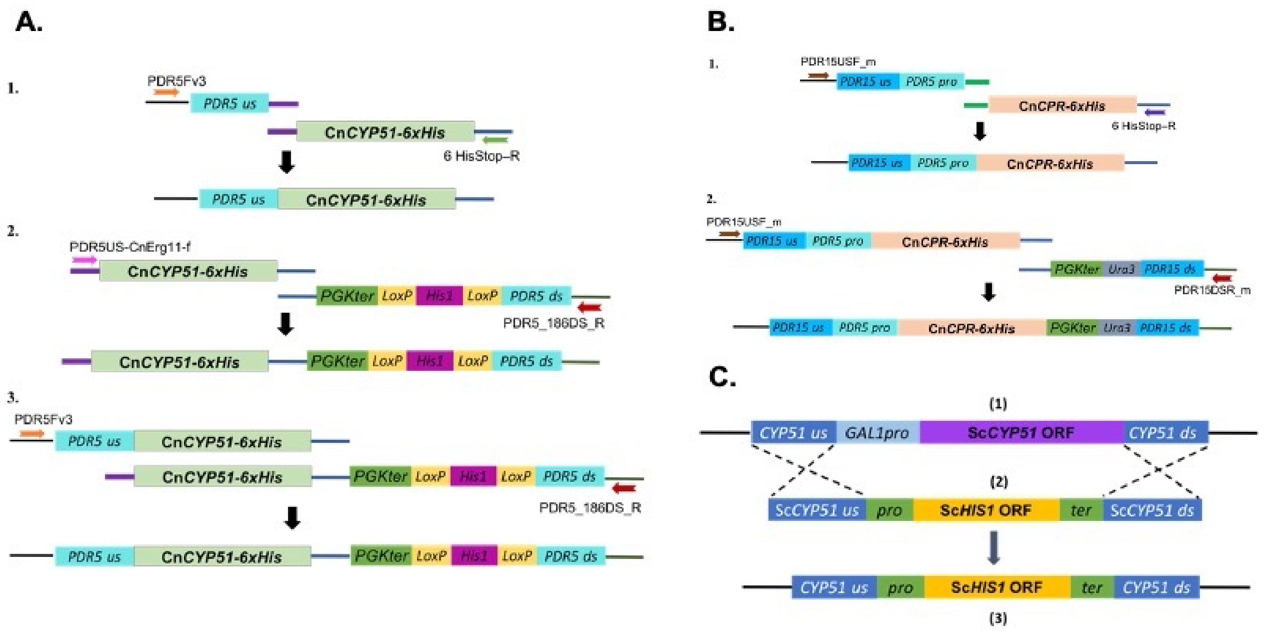

Figure S1. Construction of recombinant strains. A. PCR-based construction of the CnCYP51-6×His transformation cassette. B. PCR-based construction of the CnCPR-6×His transformation cassette C. Homologous recombination of Sc*HIS1* disruption cassette at the Sc*ERG11* locus. The native Sc*ERG11* along with the galactose promoter was replaced by the Sc*HIS1* disruption cassette. (1) *ScERG11* in the native locus. (2) Sc*HIS1* disruption cassette. (3) Sc*HIS1* integration at the native *ERG11* locus.

A. Mass spectrometry result for CnCYP51-6×His

Digestion of CnCYP51-6×His with trypsin

MSAIIPQVQQLLGQVAQFIPPWFAALPTSVKVVIAVIGIPALVICLNVFQQLCLPRRKDLPPVVFHYIPWFGSAAYYGEDPYK
FLFECRDKYGDLFTFILMGRRVTVALGPKGNNLSLGGKISQVSAEEAYTHLTTPVFGKGVVYDCPNEMLMQQKKFIKSGLTTE
SLQSYPPMITSECEDFFTKEVGISPQKPSATLDLLKSMSELIILTASRTLQGKEVRESLNGQFAKYYEDLDGGFTPLNFMFPN
LPLPSYKRRDEAQKAMSDFYLKIMENRRKGESDHEHDMIENLQSCKYRNGVPLSDRDIAHIMIALLMAGQHTSSATSSWTLLH
LADRPDVVEALYQEQKQKLGNPDGTFRDYKYEDLKELPIMDSIIRETLRMHAPIHSIYRKVLSDIPVPPSLSAPSENGQYIIP
KGHYIMAAPGVSQMDPRIWQDAKVWNPARWHDEKGFAAAAMAQYSKAEQVDYGFGSVSKGTESPYQPFGAGRHRCVGEQFAYT
QLSTIFTYVVRNFTLKLAVPKFPETNYRTMIVQPNNPLVTFTLRNAEVKGGRHHHHHH
Protein sequence coverage is 52.34%.

Digestion of CnCYP51-6×His with chymotrypsin

MSAIIPQVQQLLGQVAQFIPPWFAALPTSVKVVIAVIGIPALVICLNVFQQLCLPRRKDLPPVVFHYIPWFGSAAYYGEDPYK
FLFECRDKYGDLFTFILMGRRVTVALGPKGNNLSLGGKISQVSAEEAYTHLTTPVFGKGVVYDCPNEMLMQQKKFIKSGLTTE
SLQSYPPMITSECEDFFTKEVGISPQKPSATLDLLKSMSELIILTASRTLQGKEVRESLNGQFAKYYEDLDGGFTPLNFMFPN
LPLPSYKRRDEAQKAMSDFYLKIMENRRKGESDHEHDMIENLQSCKYRNGVPLSDRDIAHIMIALLMAGQHTSSATSSWTLLH
LADRPDVVEALYQEQKQKLGNPDGTFRDYKYEDLKELPIMDSIIRETLRMHAPIHSIYRKVLSDIPVPPSLSAPSENGQYIIP
KGHYIMAAPGVSQMDPRIWQDAKVWNPARWHDEKGFAAAAMAQYSKAEQVDYGFGSVSKGTESPYQPFGAGRHRCVGEQFAYT
QLSTIFTYVVRNFTLKLAVPKFPETNYRTMIVQPNNPLVTFTLRNAEVKGGRHHHHHH
Protein sequence coverage is 80.94%.

B. Mass spectrometry result for CnCPR-6×His

Digestion of CnCPR-6×His with trypsin.

MLSAVDIVIITLTVALPLLYFFRESLPFIGGKTRAAAPHAAVANKANVDEGDPSDFVGKMTRANKRCVIFYGSQTGTAEEYAI
RLAKEAKSRYGLSSLVCDPEEYEMSLLDQVPEDACVIFVMATYGEGEPTDNANAMMELLQEPEPEFSQGGSTLENLNYVIFGL
GNRTYEFYNEVAKKLDKRLTELGAKRIGERGEGDDDKSMEEDYLAWKDLMWTDFAERMGVEEGGAGDVPDFVVKELHDHSPEK

VYHGELSSRALLASASGTNTPVGAYGVKNPYPAPVLASKELFAVGGDRNCIHIEFDITGTGMTYQHGDHVGIWPSNSDVEVDR
MLAVLGLAASGRRQAIVDIESLDPALAKVPFPTPATYDAIFRHYLDISAVASRQTIAFLARYAPSEAAREKLTRWGTDKEAYA
NEIDGPALKLAEVLQAASNDSTEPPFASQTVWPIPFDRIVSSVPRLQPRYYSISSSSKLHPNAIHVTAVVLKYQPTVSPPHHH
EPRWVFGLSTNFILNVKMAHSGENTPVEGDVSQVSMKKVPSYKLAGPRGHYVKENVYKVPIHVRRSTFRLPTSPKVPIIMIGP
GTGVAPFRGFVQERIALARKAIDKNGPDALKDWAPMYLFYGCRRADEDFLYREEWPRYEQELKGVFRMKVAFSREMKKPDGSK
VYVQDLIHDLASELAPLILEKRAYIYICGDAKNMSKAVEERLMEMLGAGKGGSAAVEGAKELKMLKERNRLMTDVWSGGRHHH
HHH

Protein sequence coverage is 78.4%.

Digestion of CnCPR-6×His with chymotrypsin.

MLSAVDIVIITLTVALPLLYFFRESLPFIGGKTRAAAPHAAVANKANVDEGDPSDFVGKMTRANKRCVIFYGSQTGTAEEYAI
RLAKEAKSRYGLSSLVCDPEEYEMSLLDQVPEDACVIFVMATYGEGEPTDNANAMMELLQEPEPEFSQGGSTLENLNYVIFGL
GNRTYEFYNEVAKKLDKRLTELGAKRIGERGEGDDDKSMEEDYLAWKDLMWTDFAERMGVEEGGAGDVPDFVVKELHDHSPEK
VYHGELSSRALLASASGTNTPVGAYGVKNPYPAPVLASKELFAVGGDRNCIHIEFDITGTGMTYQHGDHVGIWPSNSDVEVDR
MLAVLGLAASGRRQAIVDIESLDPALAKVPFPTPATYDAIFRHYLDISAVASRQTIAFLARYAPSEAAREKLTRWGTDKEAYA
NEIDGPALKLAEVLQAASNDSTEPPFASQTVWPIPFDRIVSSVPRLQPRYYSISSSSKLHPNAIHVTAVVLKYQPTVSPPHHH
EPRWVFGLSTNFILNVKMAHSGENTPVEGDVSQVSMKKVPSYKLAGPRGHYVKENVYKVPIHVRRSTFRLPTSPKVPIIMIGP
GTGVAPFRGFVQERIALARKAIDKNGPDALKDWAPMYLFYGCRRADEDFLYREEWPRYEQELKGVFRMKVAFSREMKKPDGSK
VYVQDLIHDLASELAPLILEKRAYIYICGDAKNMSKAVEERLMEMLGAGKGGSAAVEGAKELKMLKERNRLMTDVWSGGRHHH
HHH

Protein sequence coverage is 86.4%

Figure S2. Identification of the recombinant proteins by mass spectrometry. Sequences identified in excised SDS-PAGE gel bands by mass spectrometry are highlighted in grey.

References

1. Lamping E, Monk BC, Niimi K, Holmes AR, Tsao S, Tanabe K, et al. Characterization of three classes of membrane proteins involved in fungal azole resistance by functional hyperexpression in *Saccharomyces cerevisiae*. *Eukaryot Cell*. 2007;6:1150-65.
2. Monk BC, Tomasiak TM, Keniya MV, Huschmann FU, Tyndall JD, O'Connell JD, 3rd, et al. Architecture of a single membrane spanning cytochrome P450 suggests constraints that orient the catalytic domain relative to a bilayer. *Proc Natl Acad Sci U S A*. 2014;111:3865-70.
3. Sagatova AA, Keniya MV, Wilson RK, Sabherwal M, Tyndall JD, Monk BC. Triazole resistance mediated by mutations of a conserved active site tyrosine in fungal lanosterol 14alpha-demethylase. *Sci Rep*. 2016;6:26213.
4. Monk BC, Keniya MV, Sabherwal M, Wilson RK, Graham DO, Hassan HF, et al. Azole Resistance Reduces Susceptibility to the Tetrazole Antifungal VT-1161. *Antimicrob Agents Chemother*. 2019;63:e02114-18.
